# Augmented reality for rhinoplasty: 3D scanning and projected AR for intraoperative planning validation

**DOI:** 10.1049/htl2.12116

**Published:** 2024-12-17

**Authors:** Martina Autelitano, Nadia Cattari, Marina Carbone, Fabrizio Cutolo, Nicola Montemurro, Emanuele Cigna, Vincenzo Ferrari

**Affiliations:** ^1^ Department of Information Engineering University of Pisa Pisa Italy; ^2^ EndoCAS Center University of Pisa Pisa Italy; ^3^ Department of Neuroscience Azienda Ospedaliero Universitaria Pisana (AOUP) Pisa Italy; ^4^ Plastic Surgery and Microsurgery Unit Department of Translational Research and New Technologies in Medicine and Surgery University of Pisa Pisa Italy

**Keywords:** augmented reality, computer vision, surgery

## Abstract

Rhinoplasty is one of the major surgical procedures most popular and it is generally performed modelling the internal bones and cartilage using a closed approach to reduce the damage of soft tissue, whose final shape is determined by means of their new settlement over the internal remodelled rigid structures. An optimal planning, achievable thanks to advanced acquisition of 3D images and thanks to the virtual simulation of the intervention via specific software. Anyway, the final result depends also on factors that cannot be totally predicted regarding the settlement of soft tissues on the rigid structures, and a final objective check would be useful to eventually perform some adjustments before to conclude the intervention. The main idea of the present work is the using of 3D scan to acquire directly in the surgical room the final shape of the nose and to show the surgeon the differences respect to the planning in an intuitive way using augmented reality (AR) to show false colours directly over the patient face. This work motivates the selection of the devices integrated in our system, both from a technical and an ergonomic point of view, whose global error, evaluated on an anthropomorphic phantom, is lower than ± 1.2 mm with a confidence interval of 95%, while the mean error in detecting depth thickness variations is 0.182 mm.

## Graphical abstract

The main idea of the present work is the using of 3D scan to acquire directly in the surgical room the final shape of the nose and to show the surgeon the differences respect to the planning in an intuitive way. This is achieved by using projected augmented reality to project false colours directly onto the patient's face.

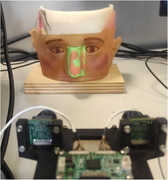

## 1 INTRODUCTION

Rhinoplasty comes from the two terms rhis and plássein, in ancient Greek, which mean nose and form respectively [1]. It is a surgical procedure that changes the external appearance and functional characteristics of the nose through the reshaping of the skin and underlying osteo‐cartilaginous structures [2].

Originally developed as a reconstructive technique due to the practice of amputation, rhinoplasty has evolved in modern times to meet the aesthetic demands of beauty. As the central feature of the face, the nose plays a crucial role in facial symmetry and proportion, which are key elements in the perception of beauty [3]. However, this does not mean that the surgery is exclusively a cosmetic procedure because, regardless of the motivation for the surgery, the role of the surgeon is also to preserve, or if necessary improve, the function of the organ and to ensure proper airflow.

Nowadays, rhinoplasty is one of the plastic surgical procedures most popular and requested in the world [4] and it is generally performed modelling the internal bones and cartilage, using (when possible) a closed approach to reduce the damage to soft tissue. The final shape of the nose is determined by how the soft tissues settle over the remodelled rigid structures.

In general, facial plastic surgery is a constantly evolving field, with continuous innovative advances in surgical techniques and additional cosmetic technologies [5]. In rhinoplasty, as underlined in [6], an optimal planning, achievable thanks to advanced acquisition of 3D images and thanks to the virtual simulation of the intervention via specialized software, is fundamental. In fact, virtual simulation helps to understand the feasibility of a technique and preliminarily evaluate the result of the treatment and, furthermore, by its combination with digital images, it may be helpful to explain to the patient the procedure that will be performed operating site.

The implementation of the planning in the surgical room requires to perform tracking and registration, which in general means to determine the exact position of skin markers, in accordance with the virtual preoperative plan, and to locate the surgical instruments used for osteotomy. According to what is reported in [6, 7], these aspects made it possible to avoid performing blind manoeuvres and to ultimately obtain precise control of the bone and cartilage cutting.

Anyway, the final result depends also on factors that cannot be totally predicted regarding the settlement of soft tissues on the rigid structures [8], and a final objective check would be useful to eventually perform some adjustments before to conclude the intervention.

In this context, the introduction of augmented reality (AR) could be particularly valuable. This technology, which is increasingly being adopted in medical field, converts preoperative 3D images into virtual data that aids plastic surgeons during procedures [9, 10, 11, 12].

However, as highlighted in several systematic reviews [13, 14, 15] there is a notable gap in studies and devices dedicated to the use of AR in plastic surgery. For preoperative planning, AR has been shown to significantly enhance the accuracy of osteotomies, especially in mandibular angle surgeries [16], and it is also beneficial in the treatment of orbital hypertelorism [17]. Additionally, AR has reduced flap harvest times in DIEP (deep inferior epigastric perforator) and SCIP (superficial circumflex iliac artery perforator) flaps, proving to be superior or at least comparable to Doppler ultrasound for vessel identification [18, 19, 20].

In rhinoplasty, an interesting work is presented by Neves et al. [21] in which they demonstrated that the use of holographic AR allows for improved external approaches to the frontal sinus. This is due to better visualization of target structures during the surgical procedure. Instead, Huang et al. [22] proposed a surgical plan utilizing augmented reality (AR) combined with guide template technology to correct nasal deformities, evaluating its feasibility and clinical efficacy. The AR‐guided surgery resulted in satisfactory nasal shapes, with a maximum error ranging from 2.24 to 3.10 mm and a mean error between 0.54 and 0.65 mm when comparing preoperative plans to postoperative outcomes.

The main idea of the present work is the using of 3D scan to acquire directly in the surgical room the final shape of the nose and to show the surgeon the differences respect to the planning in an intuitive way using augmented reality to show false colours directly over the patient face. The paper motivates the selection of the devices integrated in our system both from a technical and an ergonomic point of view and report the results of a validation study performed on an anthropomorphic phantom to validate the total system precision and accuracy.

## 2 MATERIALS AND METHODS

### 2.1 AR display choice

Currently, there is a growing interest about AR in surgery [23] and many systems are already available. In general, considering the specific anatomy and intervention workflow, the system and its functionalities should be intuitive, efficacy, accurate and ergonomic. There are some technological key points to take into account for the implementation of an AR system [24] in order to obtain all of the above‐mentioned goals.

One of the first considerations is the choice of display type, which significantly impacts both ergonomics and accuracy. Wearable AR systems based on head‐mounted displays (HMDs) are deemed as an ergonomic and effective solutions for guiding interventions performed under the surgeon's direct vision. This is due to their ability to preserve the user's egocentric perspective [25].

Anyway, to maximize the ergonomics we preferred a spatial projector‐based solution avoiding the need for head‐mounted devices [26]. Given their esocentric point of projection, in projector‐based AR systems the overlay accuracy is affected by the user point of view in case of virtual information related to deep, not exposed anatomical structures [27]. However, in our application the information to be displayed is consistent with the current nose surface, thus without any overlay inaccuracy due to the spatial displacement between the projector and the user [28].

Given the small area of projection required, we decided to use a mini‐projector that could be positioned close to the patient's face, thereby maximizing the spatial resolution of the AR view. Additionally, given the short distances involved in the procedure, we preferred a laser projector to always obtain an in focus image.

### 2.2 3D scan implementation

There are many available technologies to perform 3D of surfaces without contacts. Available devices based on time delay approaches (as Lidar), to the best of our knowledge, do not offer the required sub‐millimetric precision along all three directions, and for this reason we opted for a stereo‐photogrammetric approach, which can be implemented integrating many already available devices tuning the design on the base of the specific requirements. Passive stereo‐photogrammetric approaches, based on point disparities search over the two images and cosine law (triangulation), can be not robust in case of low texture surfaces, as often some areas of the face. However, in our case, the natural texture can be improved projecting random dots onto the face using the projector, trying to achieve more accurate disparity detection [29]. Furthermore, a camera‐based 3D scan system offer also images that are useful to perform the registration of the planning of the patient as described in Section 2.6.

We selected the couple of cameras and their arrangement in order to reduce the system's overall footprint and to obtain the required sub‐millimetric precision, whose main error is along the depth direction:

(1) $$\Delta z=\frac{d^{2}}{fb}$$

where *d* is disparity of corresponding points in the two images, *f* is the focal length of the cameras, and *b* is the baseline distance between the cameras.

### 2.3 Integrated system

**FIGURE 1:**
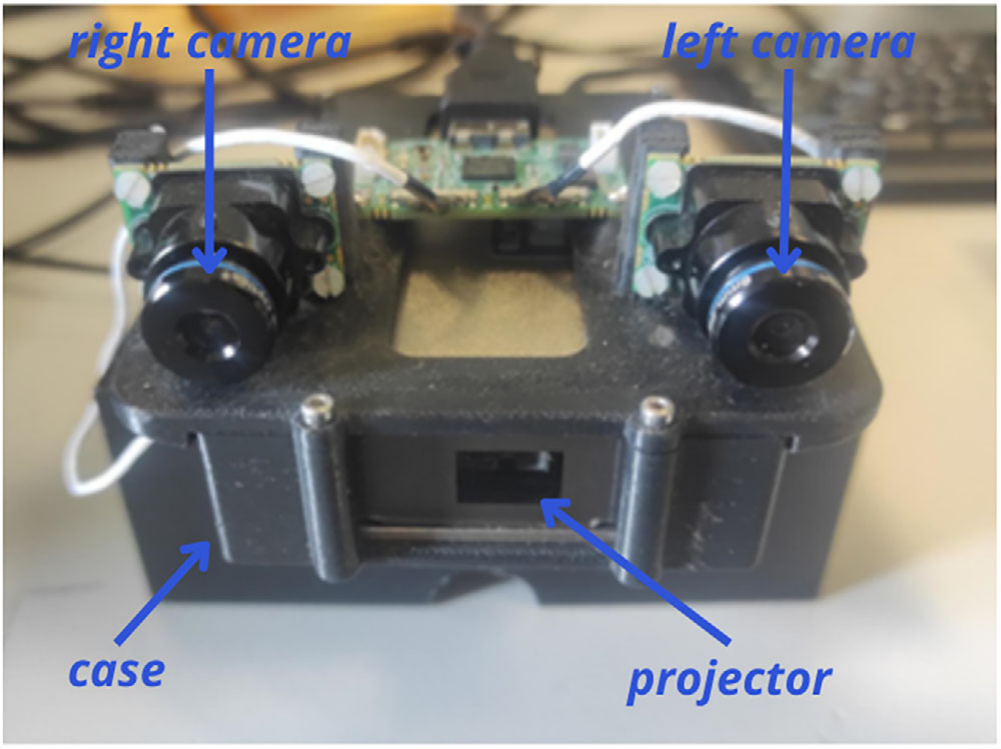
Integrated system with the 2 cameras, named right and left camera, and the projector, all assembled in the 3D printed case.

Given the motivations described in Sections 2.1 and 2.2 we selected a couple of Leopard LI‐OV580 cameras whose specifications are reported in Table 1 and an AnyBeam HD301M1 projector whose specifications are reported in Table 2. The devices are assembled in an ad‐hoc designed and 3D printed case as depicted in Figure 1.

**TABLE 1:** Leopard LI‐OV580 cameras specifications.

**Lens specifications**	
Focal length	3.10 mm ± 5%
Aperture	2.0 ± 5%
Field of view	113.2° diagonal 98.0° horizontal 58.0° vertical
TV distortion	<−10.8%
**General behaviour**	
Video resolution	2560 × 720 (2 × 1280 × 720) @ 60 fps
Sensitivity	1900mV/Lux‐s
**Optical interface**	
Resolution	2688(H) × 1520(V) (active pixels)
Pixel size	2.0 × 2.0 µm
**Electrical interface**	
Supply voltage	USB 3.0 + 5VDC power source
**Mechanical interface**	
Sizes	40.0 (L) × 26.0 (W) mm
Weight (with lens and 2 cables)	∼31 g

**TABLE 2:** AnyBeam HD301M1 projector specifications.

**Specifications and features**	
Imaging technology	MEMS laser scanning
Resolution	720P @ 60 fps
Brightness	ANSI 30 lumens ± 10%
Contrast ratio	80,000:1
I/O interface	HDMI
Rated voltage/current	5VDC/1.5 A
Dimensions (W × H × D)	∼103 × 60 × 19 mm
Weight	∼140 ± 10%

**FIGURE 2:**
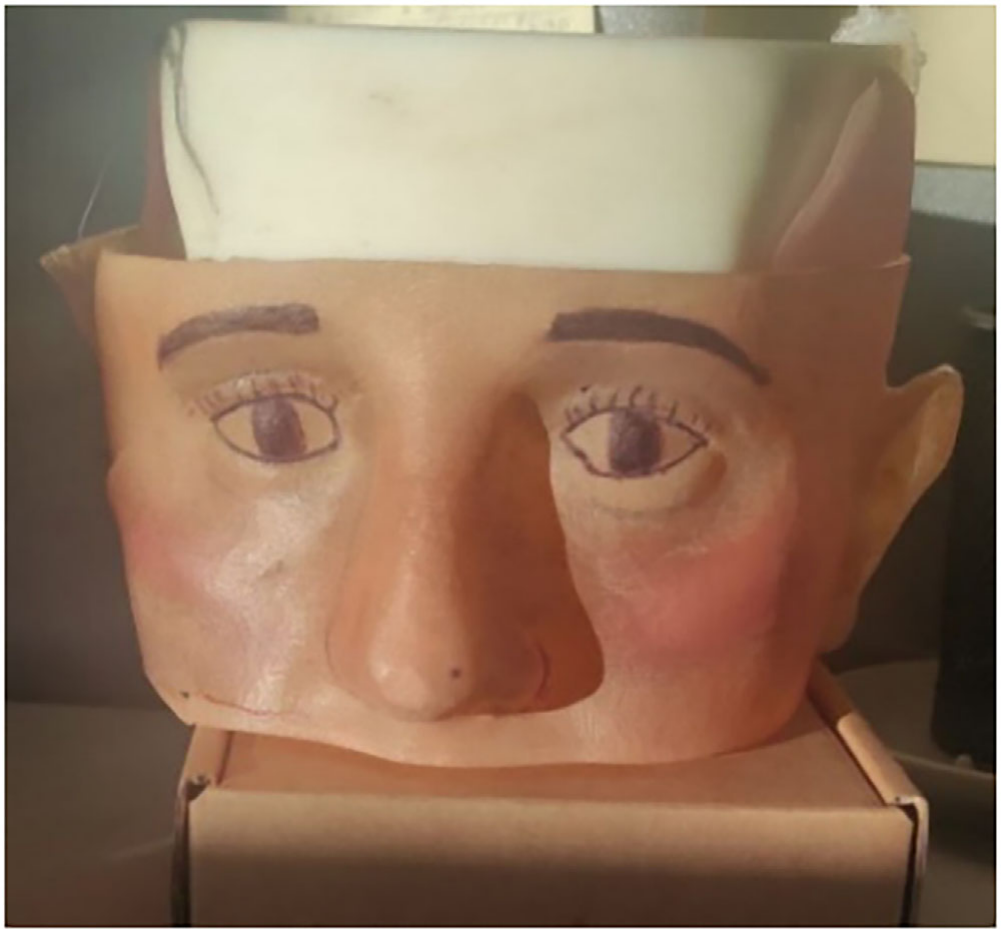
Anthropomorphic phantom.

All test are performed using an anthropomorphic phantom manufactured with 3D printing technology (the PolyJet anatomy printer, Stratasys Inc, CA). The phantom is designed to replicate cranial bones with plastic and facial tissues with silicone. Facial features such as eyes, eyelashes, eyebrows and variable skin pigmentation are drawn on the face to make it as realistic as possible (Figure 2). This attention to detail ensures that the facial texture captured by the cameras closely resemble those of a real patient, which is essential for accurate 3D scanning and reconstruction.

**FIGURE 3:**
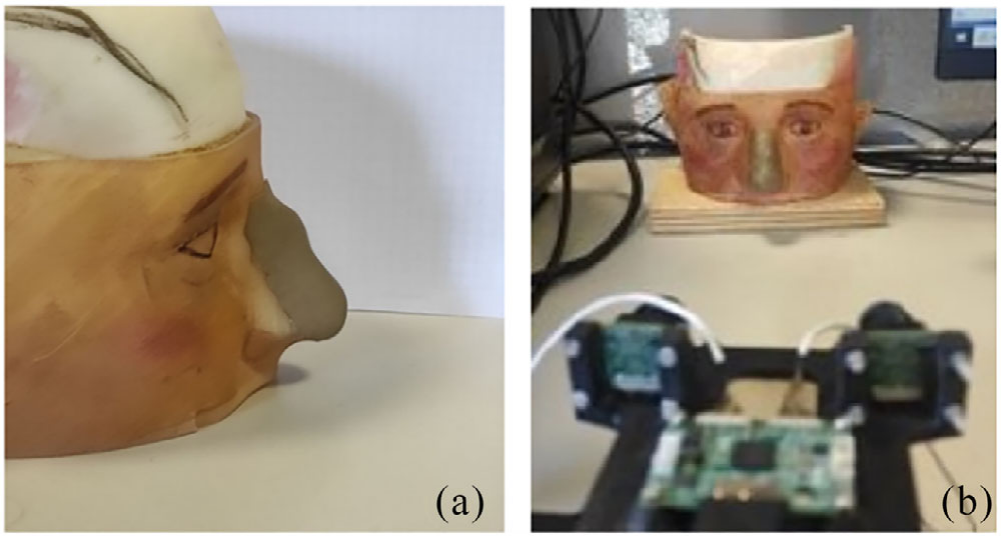
Modified phantom model with plasteline in side view (a) and in camera viewpoint during acquisition (b).

The STL file of this phantom is available, and from it, a 3D point cloud is extracted. This point cloud is used as the planning surface, which, in our experiments, represents the final goal that the surgeon aims to achieve through the surgical procedure. For comparison purpose, the 3D surface of the modified phantom, which includes an altered nose created with pasteline, is acquired by scanning (as described in Section 2.5) the phantom model, (Figure 3). This modified surface is used to evaluate the accuracy of the surgical procedure against the planned outcome.

### 2.4 Cameras and projector calibration

To perform the 3D scan, both intrinsics and extrinsics parameters of the couple of cameras are required, which are determined using the well‐known Zhang [30] method using OpenCV routines and images of a structured planar chessboard [31].

**FIGURE 4:**
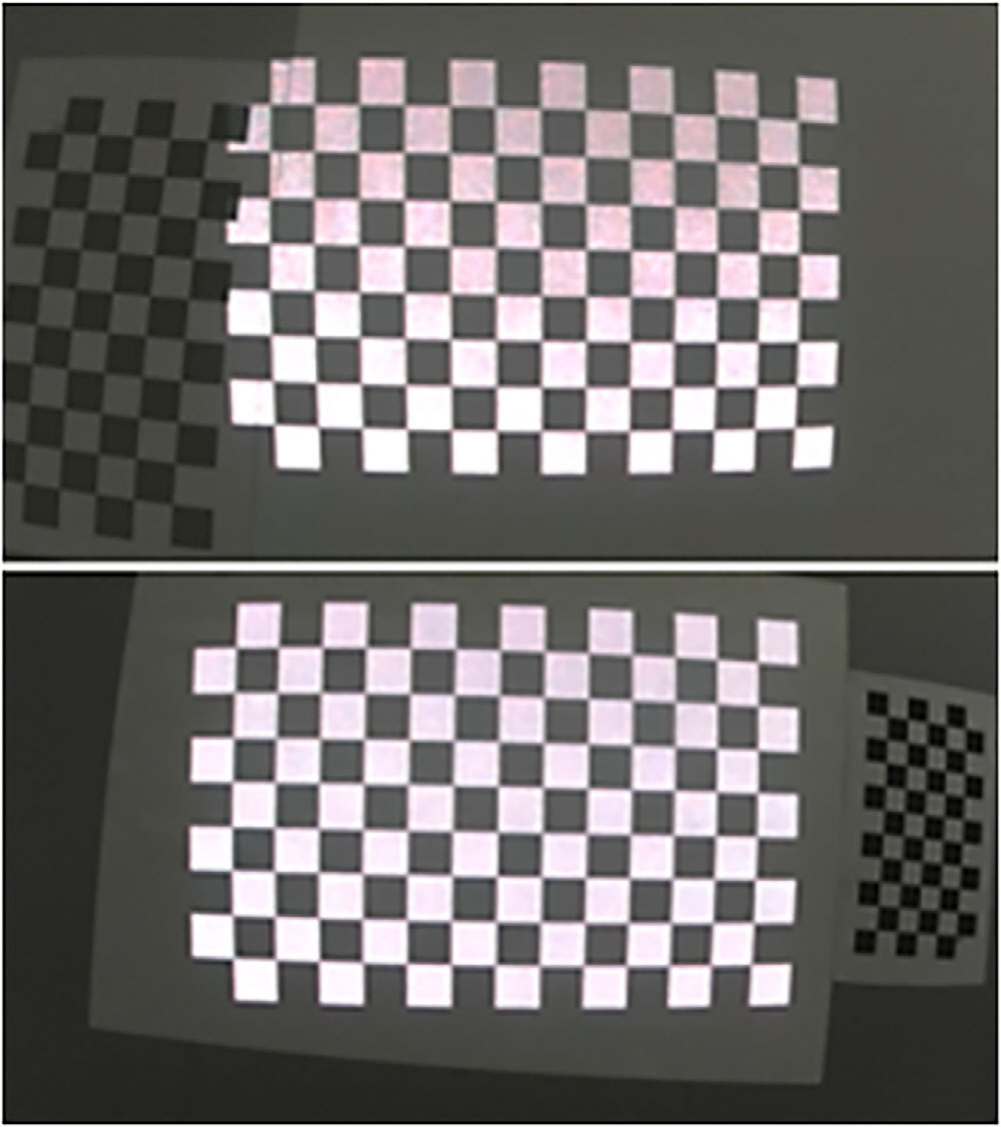
Several images were captured by the cameras for projector calibration, each containing both a real chessboard and a projected virtual chessboard, positioned in different relative orientations.

Once the cameras are calibrated, another calibration between the camera and the projector is required to accurately project the 3D AR information, obtained starting from the planning and the 3D scan, onto the patient's nose. For this purpose, the projector is modelled as a “reverse camera”, which rather than acquiring, projects light rays from a 2D image in the 3D space. The goal of this calibration is to identify the 3D points of the projected pattern corresponding to the 2D points of the projected image and thus obtain the intrinsic calibration parameters of the projector and extrinsic in respect to the camera. The calibration is performed by capturing a set of images (Figure 4) that include both a physical planar chessboard and a projected virtual chessboard. We use a state‐of‐the‐art approach [32] that is based on the identification of corners of the chessboards in the reference system of the camera by applying the ray‐plane intersection that we previously implemented in Matlab.

### 2.5 Video‐based 3D scan

**FIGURE 5:**
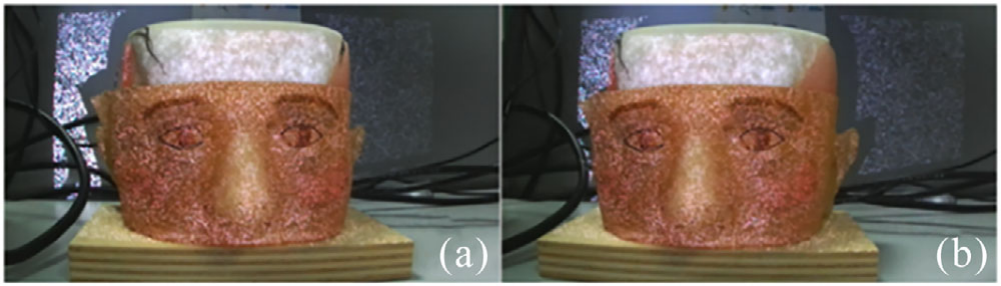
A couple of camera images acquired on the phantom with projected random dots to improve the disparity map. (a) left image, (b) right image.

The 3D scan of the face is obtained with a classical stereophotogrammetric approach, implementing through a C++ application built on the top of the OpenCV library. To find the image disparities, we used the block matching algorithm with a window size of 7 × 7 pixels. To minimize holes in the disparity map we projected onto the face random dots [29], as illustrates in Figure 5.

The stereoscopic cameras calibration data are used both to speed up the disparity search, by using rectified images, than to generate the depth map.

The result is a sparse 3D point cloud and, given some difficulties encountered in the reconstruction especially of the lateral walls and, sometimes, of the dorsum of the nose, we applied a refinement using self‐organized maps [33].

### 2.6 Surgical planning registration

**FIGURE 6:**
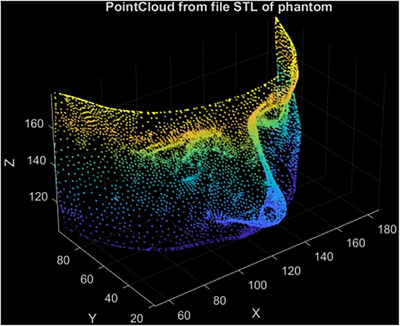
Example of surgical planning consisting in the vertex of the mesh representing the desired result.

The surgical plan (Figure 6), consisting in the vertex of the mesh representing the desired result, is registered with the 3D scanning of the patient after surgery to compare the results against the plan.

**FIGURE 7:**
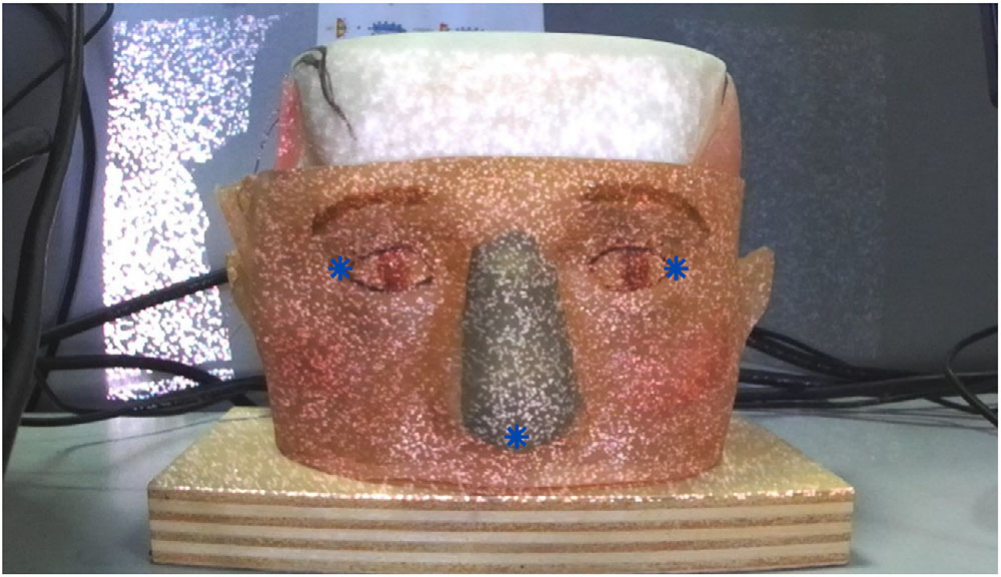
The three selected points (* in blue) by the user to initialize the ICP registration.

For this purpose, we opted for an ICP (iterative closest point) surface‐based registration between the planned and the actual surface. The registration algorithm and user interface are implemented in Matlab, and the ICP registration is initialized with an initial point‐based registration. Specifically, the user selects three points (eye angles and nose tip as shown in Figure 7) in the acquired images and the algorithm determines their 3D position. Then it calculates the roto‐translation with respect to the corresponding three selected points in the planning surface using the least squares method.

### 2.7 AR false colours maps

**FIGURE 8:**
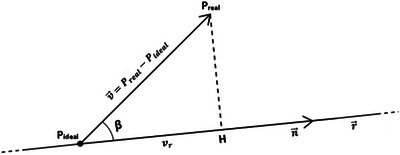
Explanatory diagram for calculating the *P*_real_ current point nearest to the line of the ideal point. *v*_r_ is the orthogonal projection of the vector $\vec{v}$ along the line r containing the normal $\vec{n}$.

The goal of the AR visualization is to show in false colours the depth differences between the current patient's face and the planned model. These differences are calculated from comparing point clouds acquired from a 3D scan of the face to those of the planned point cloud along the nose's normal axis. In particular, each real point *P*_real_ in the current point cloud is coupled with the ideal point *P*_ideal_ in the planned point cloud whose normal direction is closest to said real point (Figure 8). The difference vector $\vec{V}$ is then calculated as follows:

(2) $$\vec{V}=P_{\mathrm{real}}-P_{\mathrm{ideal}}$$

and its oriented length in respect to the normal to the face: positive values indicate points where *P*_real_ is above the planned surface, while negative values indicate points where it is below.

**FIGURE 9:**
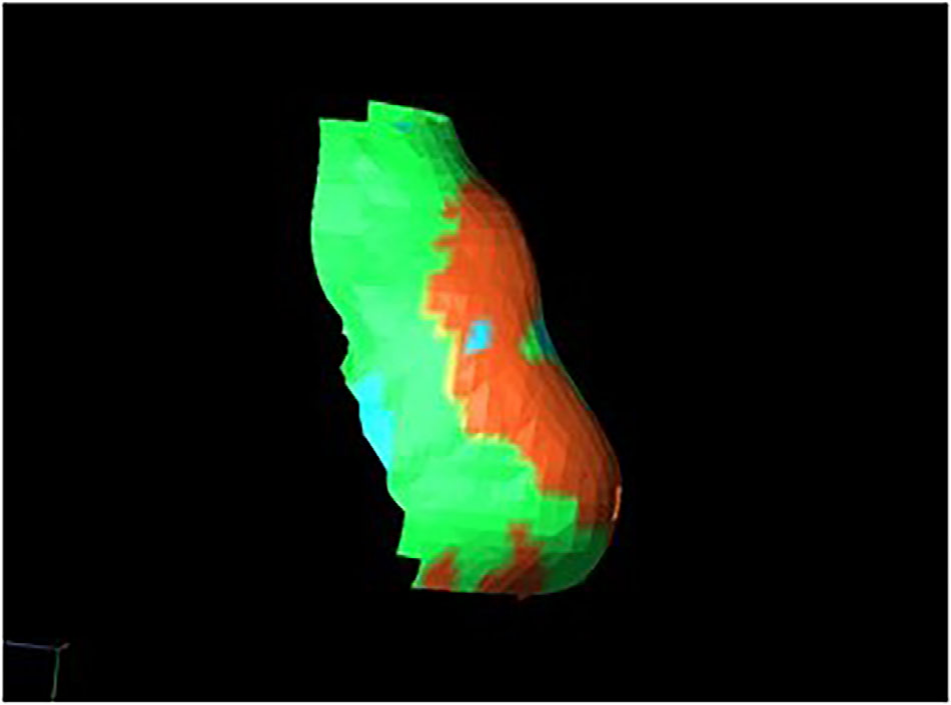
Coloured 3D mesh whose 2D rendering is overlayed bye means the projector over the nose.

Our Matlab application uses these depth errors to create a 3D mesh with the same morphology of the current nose, but colour‐coded with a false colour map. The depth error to colour mapping can be done selecting colours and thresholds as preferred by the surgeon. For example, areas where the current nose exceeds, falls short, or aligns with the ideal nose can be represented in red, blue, and green, respectively (Figure 9).

**FIGURE 10:**
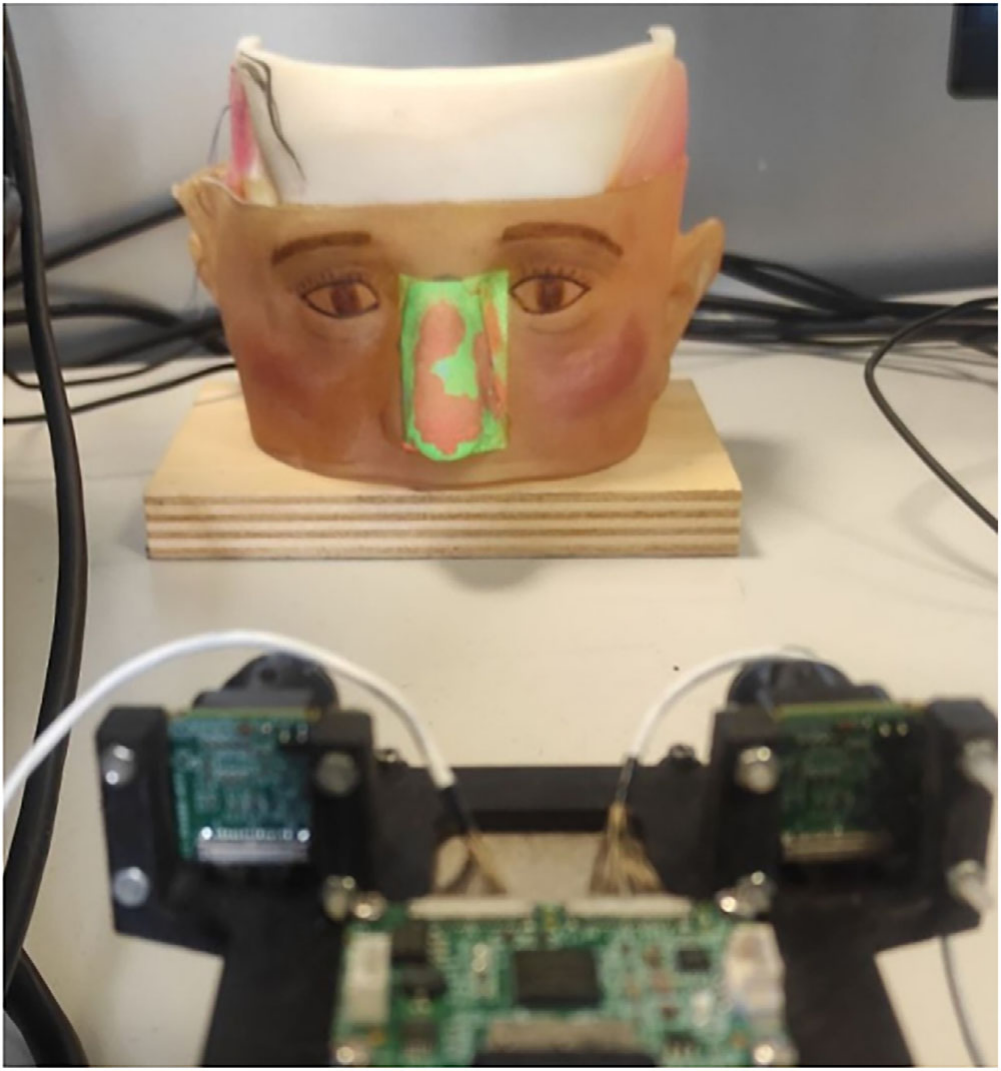
Projection of the false colour map over to nose: green areas are in the depth threshold interval; red areas are over the threshold.

Finally, to project this 3D mesh onto the patient's nose, the application first converts it into a 2D image. This is achieved by rendering the 3D mesh using a virtual camera that is calibrated to match the real projector's parameters. To ensure a precise overlay, the virtual camera's pose is aligned with the projector's pose relative to the camera system. Since both the current point cloud and the 3D coloured mesh are represented in the left, rectified camera's coordinate system, this alignment is crucial for accuracy. Finally, the rendered 2D image undergoes distortion correction based on the distortion parameters obtained during the projector calibration phase. This step compensates for any optical distortions introduced by projector lens, ensuring a coherent overlay, as shown in Figure 10.

### 2.8 Precision validation test and false colours map threshold setting

To avoid noise in the false colours map visualization, the threshold must be set on the base of the system precision, which is globally influenced by the 3D scan precision, the projector calibration and the registration result.

To estimate the global precision of our system, we measured the depth error in a controlled setup. We performed 3D scans of the phantom with and without additional material applied over the nose, simulating a wrong surgical execution.

**FIGURE 11:**
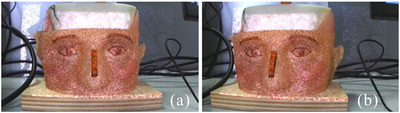
A couple of camera images acquired on the phantom with a strip over the nose to simulate a not well executed intervention. (a) left image, (b) right image.

In particular, the tests involved the positioning of strips with known thickness on the phantom's nose (Figure 11), after which the usual operations of 3D scan, registration and calculation of the false colours map were carried out, to analyse the distribution of distance values at the centre points of the added thicknesses.

It is important to note that the strips were placed on the dorsum of the nose for two key reasons. Firstly, as discussed in Section 2.5, the system often struggles to achieve accurate 3D reconstruction of the side walls of the nose due to their vertical orientation. In contrast, the dorsum of the nose consistently yields a more reliable reconstruction. This ensures that the subsequent distance calculations are more accurate and trustworthy, which might not be the case if the strips were placed on other, less reliably reconstructed areas of the nose.

Secondly, the verticality of the side walls not only complicates the 3D reconstruction but also exacerbates errors during the reprojection of the false colour map. By focusing on the dorsum of the nose, we minimize the impact of these verticality‐related errors, resulting in a more reliable estimate of global system accuracy.

The strips were made from a calibrated sheet of EVA rubber with a thickness of 2 mm. For further tests, we created thicker layers by stacking and adhering two or three strips using double‐sided tape, resulting in thicknesses of 4 and 6 mm, respectively.

Three consecutive acquisitions were performed for each strip thickness (0, 2, 4, and 6 mm), obtaining a total of 12 tests.

The distances between the 3D point clouds with and without the EVA strips were then calculated. To ensure the accuracy of our measurements and avoid unrealistic side effects at the strip margins, distance calculations were focused only on points near the strip centre line. This approach provided a reliable estimation of the system's precision, ensuring that the threshold for the false colour map visualization is appropriately set.

## 3 RESULTS AND DISCUSSION

**FIGURE 12:**
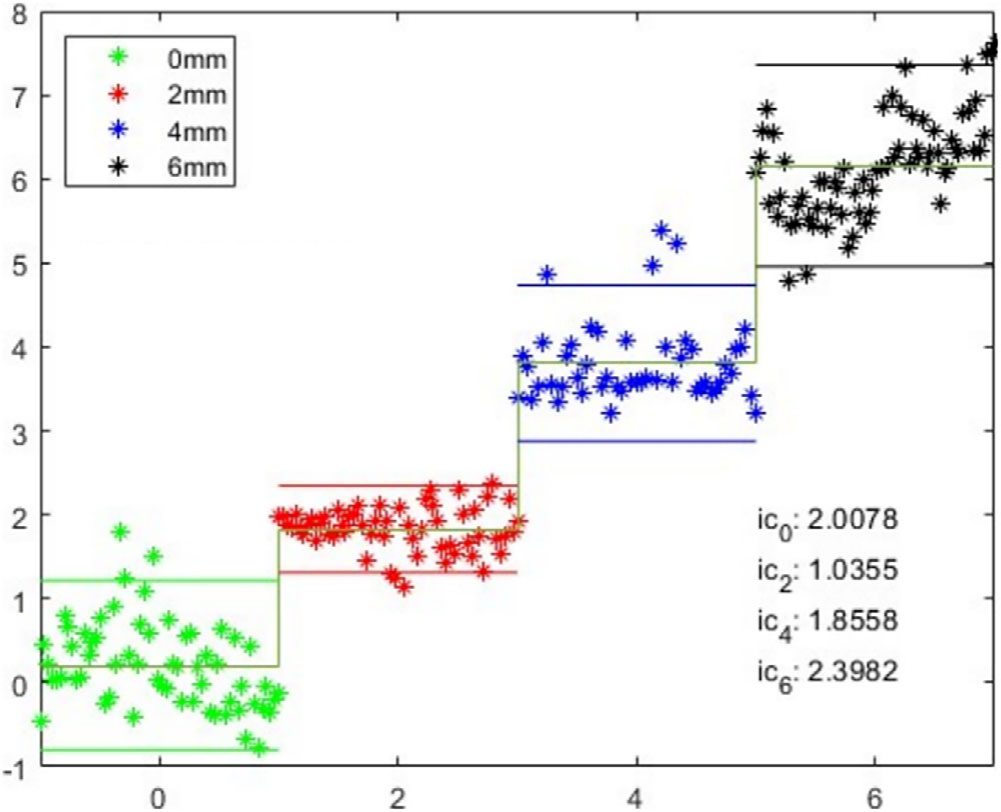
Depth errors with data aggregated for each strip thickness and the corresponding 95% confidence interval.

The results of the tests described in Section 2.8 are depicted in the graph in Figure 12, with data aggregated by strip thickness. For each point in the current cloud is reported the distance in respect to the planned one (along the nose axis).

Given a Gaussian distribution of these data, as confirmed by the Kolmogorov–Smirnov test, we calculated the 95% confidence interval for each strip thickness. These results are shown in the same Figure 12, and the mean estimated depth increases are summarized in Table 3.

**TABLE 3:** Mean of data for each strip thickness.

Real increased thickness (mm)	Mean estimated increasement (mm)	Absolute mean (mm)
0	0.206	0.206
2	1.832	0.168
4	3.804	0.196
6	6.158	0.158

In general, the confidence interval provides an indication of the precision and variability of the measure, and it can be used to establish the threshold for the false colour map visualization.

In our particular case, with working distances between the device and the phantom exceeding 25 cm, the cameras focal length of about 1500 pixels, and their baseline distance of 60 mm, the theoretical limit of depth resolution, considering a disparity of just one pixel, is 0.7 mm (from Equation 1). This resolution is related to reliability with which the correspondences between the two images were identified, which in turn depends on the inhomogeneity of the texture of the object and by the block matching algorithm used. In other words, depth differences smaller than this theoretical limit would be undetectable, and consequently the threshold values will be set above this theoretical limit to avoid showing wrong information to the surgeon and/or a noisy AR colour map.

Following this reasoning, given the obtained experimental results we must take into account the maximum of the confidence intervals. His value is 2.4 mm and, therefore, the precision provided by the system could be defined as approximately equal to ±1.2 mm, which will therefore be the minimum interval between the thresholds in the depths to colours mapping. In addition, the mean error in detecting depth variations was calculated to be 0.182 mm.

This work introduces significant innovation in the field of rhinoplasty, particularly by addressing the notable gap in the application of AR technology within this surgical context. As highlighted in the introduction, there is a relatively limited number of studies exploring the use of augmented reality in plastic surgery [9, 10, 11, 12]. Most of the existing research focuses on evaluating the AR accuracy in organ identification and visualization, overlay onto the patient [16, 17, 18, 19, 20, 21].

Our work outlined a new implementation of augmented reality for real‐time modelling of the nose, which supports and enables the surgeon to improve his performance in the operating room. This approach ensures, early as the intraoperative phase, that the patient achieves the planned esthetical outcome, whereas such confirmation often only becomes possible long after surgery in the postoperative phase. Another significant innovation, from a sustainability perspective, is that our AR system optimizes medical resources by reducing the likelihood of complications and minimizing the need for further surgical revisions, thus improving the efficiency of the healthcare system.

Obviously, the current implementation could be improved, particularly by using more cameras to perform 3D scanning and a pair of projectors to get better results on the sides of the nose, which, due to their orientation, are not always fully reconstructed with the current system. These enhancements could expand the use of our system to other surgical procedures, including cosmetic, but also neurosurgical, maxillofacial, and orthopaedic surgeries.

To the best of the authors’ knowledge, no existing AR devices allow for direct comparison of preoperative plans with the final nose shape in the surgical room, enabling real‐time adjustments for more precise outcomes.

## 4 CONCLUSION

The main idea of the present work is the using of 3D scan to acquire directly in the surgical room the final shape of the nose and to show the surgeon the differences respect to the planning in an intuitive way. This is achieved by using augmented reality (AR) to project false colours directly onto the patient's face. This work motivates the selection of the devices integrated in our system, and the same reasonings can be used to implement improved versions.

The implemented system provides satisfactory results, ensuring high accuracy and opening up promising prospects for future use in the operating room. An error margin of ±1.2 mm is an excellent outcome, particularly in aesthetic procedures like rhinoplasty, where precision is crucial for achieving harmonious and natural results aligned with the patient expectations.

The deployment of an engineered version of our early prototype in the operating room could significantly benefit plastic surgeons, enhancing the overall quality of procedures and potentially increasing patient satisfaction. The system's ability to provide real‐time, accurate feedback during surgery represents a substantial advancement in the field of plastic surgery, particularly in achieving precise and predictable outcomes in rhinoplasty.

## AUTHOR CONTRIBUTIONS

Vincenzo Ferrari conceived the experiments, supervised the work, and wrote the manuscript; Martina Autelitano conceived and designed the experiments, Martina Autelitano and Nadia Cattari analysed the results and wrote the first draft; Martina Autelitano and Marina Carbone reviewed and validated the final manuscript; Martina Autelitano conducted the experiments and prepared the figures and tables; Marina Carbone, Fabrizio Cutolo, Nicola Montemurro, and Emanuele Cigna participated in conceiving the research. All authors reviewed the manuscript

## CONFLICT OF INTEREST STATEMENT

The authors declare no conflicts of interest.

## Data Availability

The data that support the findings of this study are available from the corresponding author upon reasonable request.
